# Enhancements of Cancer Cell Damage Efficiencies in Photothermal and Photodynamic Processes through Cell Perforation and Preheating with Surface Plasmon Resonance of Gold Nanoring

**DOI:** 10.3390/molecules23123157

**Published:** 2018-11-30

**Authors:** Jen-Hung Hsiao, Yulu He, Jian-He Yu, Po-Hao Tseng, Wei-Hsiang Hua, Meng Chun Low, Yu-Hsuan Tsai, Cheng-Jin Cai, Cheng-Che Hsieh, Yean-Woei Kiang, Chih-Chung Yang, Zhengxi Zhang

**Affiliations:** 1 Institute of Photonics and Optoelectronics, and Department of Electrical Engineering, National Taiwan University, No. 1, Section 4, Roosevelt Road, Taipei 10617, Taiwan; r03941030@ntu.edu.tw (J.-H.H.); heyulu198707@126.com (Y.H.); r03941097@ntu.edu.tw (J.-H.Y.); r03941025@ntu.edu.tw (P.-H.T.); r04941083@ntu.edu.tw (W.-H.H.); r04941006@ntu.edu.tw (M.C.L.); r05941091@ntu.edu.tw (Y.-H.T.); r05941084@ntu.edu.tw (C.-J.C.); r04941086@ntu.edu.tw (C.-C.H.); ywkiang@ntu.edu.tw (Y.-W.K.); 2 Institute of Micro/Nano Photonic Materials and Application, School of Physics and Electronics, Henan University, Kaifeng 475004, China; 3 Key Laboratory of Biomedical Information Engineering of Education Ministry, Institute of Biomedical Analytical Technology and Instrumentation, School of Life Science and Technology, Xi’an Jiaotong University, No.28, Xianning West Road, Xi’an 710049, Shanxi, China; zxzhang@mail.xjtu.edu.cn

**Keywords:** cell perforation, cell preheating, surface plasmon resonance, photothermal therapy, photodynamic therapy

## Abstract

The methods of cell perforation and preheating are used for increasing cell uptake efficiencies of gold nanorings (NRIs), which have the localized surface plasmon resonance wavelength around 1064 nm, and photosensitizer, AlPcS, and hence enhancing the cell damage efficiency through the photothermal (PT) and photodynamic (PD) effects. The perforation and preheating effects are generated by illuminating a defocused 1064-nm femtosecond (fs) laser and a defocused 1064-nm continuous (cw) laser, respectively. Cell damage is produced by illuminating cell samples with a focused 1064-nm cw laser through the PT effect, a focused 1064-nm fs laser through both PT and PD effects, and a focused 660-nm cw laser through the PD effect. Under various conditions with and without cell wash before laser illumination, through either perforation or preheating process, cell uptake and hence cell damage efficiencies can be enhanced. Under our experimental conditions, perforation can be more effective at enhancing cell uptake and damage when compared with preheating.

## 1. Introduction

For many therapeutic applications, such as gene therapy, drug molecules need to be delivered into live cells. Certain viruses have been used as the carriers for such a delivery into the cytoplasm of targeted cells. However, potential risks exist in using viruses as drug carriers. Alternatively, a physical approach, like perforation, for membrane penetration of drugs is more attractive. Such an approach is particularly useful in in vitro application. Various techniques have been developed for cell perforation. Electroporation based on the effect of a strong electric field is a widely used technique for in vitro and in vivo applications. Although it has a high transfection efficiency, usually the cell viability based on this technique is not high [1,2,3]. Sonoporation based on the effect of an ultrasound wave for generating microbubbles to perforate the cell membrane is another useful technique. Although the generated microbubbles can cause effective cell perforation, the induced shear force using ultrasound and the temperature rise can lower cell viability [4,5]. Photoporation based on laser illumination is a newly developed technique attracting much attention. In particular, with Au nanoparticles (NPs) adsorbed onto cell membrane, we can use a low-intensity laser for achieving effective perforation [6,7,8,9,10,11,12,13]. In this situation, the illumination of a defocused laser (a pulsed laser is preferred) can induce the localized surface plasmon (LSP) resonance of the Au NPs for increasing absorption and heating such that vapor bubbles can be generated. The breakup of the vapor bubbles can produce high-pressure impact onto cells for forming temporary openings of the membrane. During the opening period, a large amount of drugs, chemicals, or NPs can enter the cells. The opening is annealed after a certain period, around 30 min. Therefore, a successful perforation process requires a significant increase of cell uptake capability and a high cell surviving percentage.

Heating of a cell within a reasonable temperature range can increase the fluidity of the phospholipidic bilayer in a cell membrane such that the permeability of the membrane can be increased. In this situation, the cell uptake capability can be enhanced [14,15,16,17,18,19,20,21]. Therefore, to increase the cell uptake efficiency of Au NPs and the photosensitizer, the application of a mild heating process to cells is a useful approach. Although heating of cells through Au NP application and laser illumination for producing the photothermal (PT) effect is an effective approach for cell damage, it usually causes cell necrosis and inflammation. In certain situations, the photodynamic (PD) effect is preferred for damaging cells through apoptosis. In such a situation, we need more photosensitizer molecules taken up by cells. For this purpose, a preheating process for increasing the uptake efficiency of a photosensitizer is useful. Therefore, the preheating temperature cannot be too high to cause significant cell damage through necrosis.

Among Au NPs of different shapes, Au nanorings (NRIs) can be fabricated for achieving strong LSP resonance in the wavelength range between 1000 and 1300 nm. In this spectral range, light can penetrate into tissue the deepest [22,23,24,25]. When Au NRIs taken up by cells are excited by a light source with the wavelength close to its LSP resonance peak, the enhanced absorption can produce a strong PT effect for causing cancer cell damage [22,23,24,25]. By linking a photosensitizer to Au NRIs for cell uptake, i.e., using Au NRIs as the photosensitizer carrier, an illumination to excite the photosensitizer can result in the PD effect. In this situation, excited by a pulsed laser, the two-photon absorption of the linked photosensitizer, which can be significantly enhanced through the LSP resonance, can lead to the simultaneous generations of the PD and PT effects [24,26]. By linking the photosensitizer, AlPcS, to Au NRIs, the combined effect of PD and PT for more effective cell damage has been reported by using a 1064-nm femtosecond (fs) laser for illumination [24,25].

Increasing cell uptake capabilities of NPs and drugs is one of the keys to improving the cancer cell damaging efficiency. In this research, we use the perforation and preheating processes based on the illuminations of a defocused fs laser and a defocused continuous (cw) laser, respectively, with their wavelengths (both at 1064 nm) coinciding with the LSP resonance spectral range of Au NRIs, for increasing the cell uptake capability of Au NRIs or the photosensitizer AlPcS, and hence enhancing cell damage efficiency through the illuminations of focused lasers. The cell damaging lasers include a focused 1064-nm cw laser for generating the PT effect, a focused 1064-nm fs laser for generating both PT and PD effects, and a focused 660-nm cw laser for generating the PD effect. The PD effect can be generated only when AlPcS is taken up by cells for generating reactive oxygen species (ROS) upon appropriate light illumination. The results show that both the perforation and preheating processes can increase cell uptake efficiency and reduce the threshold laser fluence for cancer cell damage through either PT, PD, or the combined effect.

## 2. Materials and Methods

**Figure 1:**
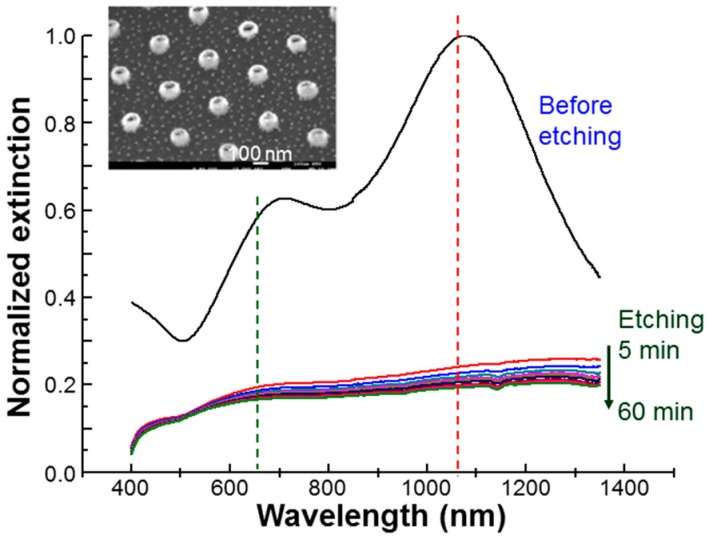
Normalized extinction spectra of the used NRI solutions before and after KI/I_2_ etching for 5–60 min. The vertical red and green dashed lines indicate the wavelengths of used lasers at 1064 and 660 nm, respectively. The insert shows the SEM image of the used Au NRIs still on substrate.

Au NRIs were fabricated first on a polymer substrate based on the techniques of nano-imprint lithography and ion-bombardment induced Au sputtering in a reactive ion etching process [22,23,24]. Briefly speaking, regularly patterned nanopillars were formed first on a polymer substrate through the processes of nano-imprint and dry etching. Then, Au was deposited onto the substrate, followed by ion bombardment for sputtering Au atoms onto the circular sidewalls of the nanopillars. After the polymer nanopillars were etched through another dry etching process, Au NRIs were formed. However, they were still attached onto the substrate. Au NRIs were then surface-modified by linking with antibodes and the photosensitizer before transferring into de-ionized water via sonication. For the Au NRI sample without linking with the photosensitizer, which is designated as sample NRI, Au NRIs were first linked with amino polyethylene glycol thiol (NH2-PEG-HS, usually abbreviated by PEG) through Au-S bonding to avoid aggregation and then were linked with a biolinker (16-mercaptohexadecanoic acid from Sigma-Aldrich, St. Louis, MO, USA) for connecting them with the anti-EGFR antibody. For the Au NRI sample linked with the photosensitizer, AlPcS, which was designated as a sample NRI-AlPcS, after the biolinker and antibody were successively connected to Au NRIs, AlPcS was connected to the linked PEG at the other end through static electric force by immersing a substrate containing ≈2 × 10^10^ Au NRI particles in a 10-mL de-ionized water solution of AlPcS (1 mM) for 24 h. After surface modification of Au NRI, Au NRIs were transferred into de-ionized water through sonication for optical characterization. The NRI solution was then condensed to ≈4 × 10^10^ cm^−3^ in particle concentration. Because the Au NRI array was regularly patterned on a substrate, we could evaluate the total number of Au NRIs on the substrate, which were then transferred into a solution. Based on the measurement of inductively coupled plasma mass spectroscopy (ICP-MS), it was estimated that 10^6^–10^7^ AlPcS molecules were linked to an Au NRI particle. Sample NRI and NRI-AlPcS solutions were used for incubating with oral cancer cells, SAS, for 12 h. In this step, the de-ionized water in the NRI or NRI-AlPcS solution was replaced with cell culture medium through a centrifuge process. It was estimated that 1000–2000 (200–300) Au NRI particles per cell were internalized into cells (adsorbed onto cell membrane). Figure 1 shows the normalized extinction spectrum of the used NRI solution (labelled by “before etching”). Here, one can see that the LSP resonance peak was located at ≈1079 nm in wavelength, which is close to the wavelength of the used cw laser at 1064 nm and fs laser also at 1064 nm (40 MHz in pulse repetition rate and 120 fs in pulse width), as indicated by the vertical red dashed line in Figure 1. Both 1064-nm cw and fs lasers could strongly excite the LSP resonance of Au NRIs for inducing enhanced absorption and hence the PT effect. Also, the strong near-field distribution at LSP resonance excited by the fs laser could enhance the two-photon absorption of AlPcS for generating ROS and hence producing the PD effect. The two-photon absorption cross-section of AlPcS at 1064 nm was as large as 12.7 GM [27]. The green vertical dashed line in Figure 1 indicates the wavelength of a cw laser at 660 nm. The 660-nm cw laser was used to excite AlPcS, which has a strong absorption in the spectral range of 650–690 nm, for generating ROS and hence the PD effect. The insert of Figure 1 shows the scanning electron microscopy (SEM) image of the used Au NRIs before liftoff with the outer diameter at ≈100 nm. Besides the NRI and NRI-AlPcS samples, we also incubated cells with NRIs and AlPcS (10 µM) separately for observing the effects of perforation and preheating. In this situation, the incubation time for NRI was also 12 h. The incubation time for AlPcS was only 30 min. This experimental condition will be referred to as NRI + AlPcS incubation.

During the process of cell incubation with either NRI or NRI-AlPcS, Au NRIs could be adsorbed onto the cell membrane or internalized into cells. To remove the effect of un-taken-up Au NRIs, cell samples were washed before perforation or preheating, and cell damaging processes, leading to the experimental condition of “post-washout”. Without this wash process, the experimental condition was designated as “pre-washout”. Under the “pre-washout” condition, the un-taken-up, adsorbed, and internalized NRIs or NRI-AlPcS existed in a cell sample. Under the condition of “post-washout,” only the adsorbed and internalized NRI or NRI-AlPcS existed in a cell sample. For identifying the perforation or preheating, and cell damage effects of the internalized Au NRIs, we used a KI/I_2_ solution (1.206 mM for KI and 0.396 mM for I_2_) to etch the Au NRIs adsorbed onto the cell membrane. After 5-min of KI/I_2_ etching, the adsorbed Au NRIs could be completely removed with cell viability maintained at a level >96%. The experimental condition after Au NRIs were etched on cell membranes was designated as “post-etching”. Under this condition, only the internalized NRIs or NRI-AlPcS existed in a cell sample. Figure 1 also shows the extinction spectra of Au NRIs after different durations of KI/I_2_ etching. One can see that 5-min of etching was long enough for dissolving NRIs such that all the LSP resonance features disappeared. Throughout this study, we used the KI/I_2_ etching duration of 5 min.

To evaluate cancer cell damage, we measured the size of an essentially circular cell damage area, which was stained using trypan blue 4 h after cells were illuminated by a circularly Gaussian-shaped laser beam. By measuring the radius of such a circular stained area, we could compute the threshold laser intensity for causing stained cell damage, which was used for evaluating the cell damage efficiency under a certain PT/PD condition [23,24,25]. The radius-dependent profile of the intensity or power density, *I*(*r*), of a Gaussian-shaped laser beam at the cell layer in a culture well can be expressed as:

(1) $$I\left(r\right)=I_{0}exp\left(-r^{2}/r_{0}^{2}\right)$$

Here, *r*_0_ is the laser beam size and *I*_0_ is the peak intensity at the beam center, which is given by $P/\left(\pi r_{0}^{2}\right)$ with *P* standing for the total laser power. By assuming that the measured radius of a circular stained area is *r_th_*, at which the laser intensity corresponds to the threshold level for causing cell damage, the threshold intensity, *I_th_*, can be evaluated through:

(2) $$I_{th}=\frac{P}{\pi r_{0}^{2}}exp\left(-r_{th}^{2}/r_{0}^{2}\right)$$

For a pulsed laser, the aforementioned intensity, power, and threshold intensity refer to the time-average values. The threshold fluence of either a cw or a pulsed laser for causing cell damage can be obtained through multiplying the evaluated threshold intensity (average threshold intensity for a pulsed laser) by the illumination duration. The determination of the circular stained area may not be very accurate because of the non-uniform distribution of cultured cells. However, this evaluation method is quite accurate for us to observe the reasonable variation trends of cell death area under different cell damage conditions.

**Figure 2:**
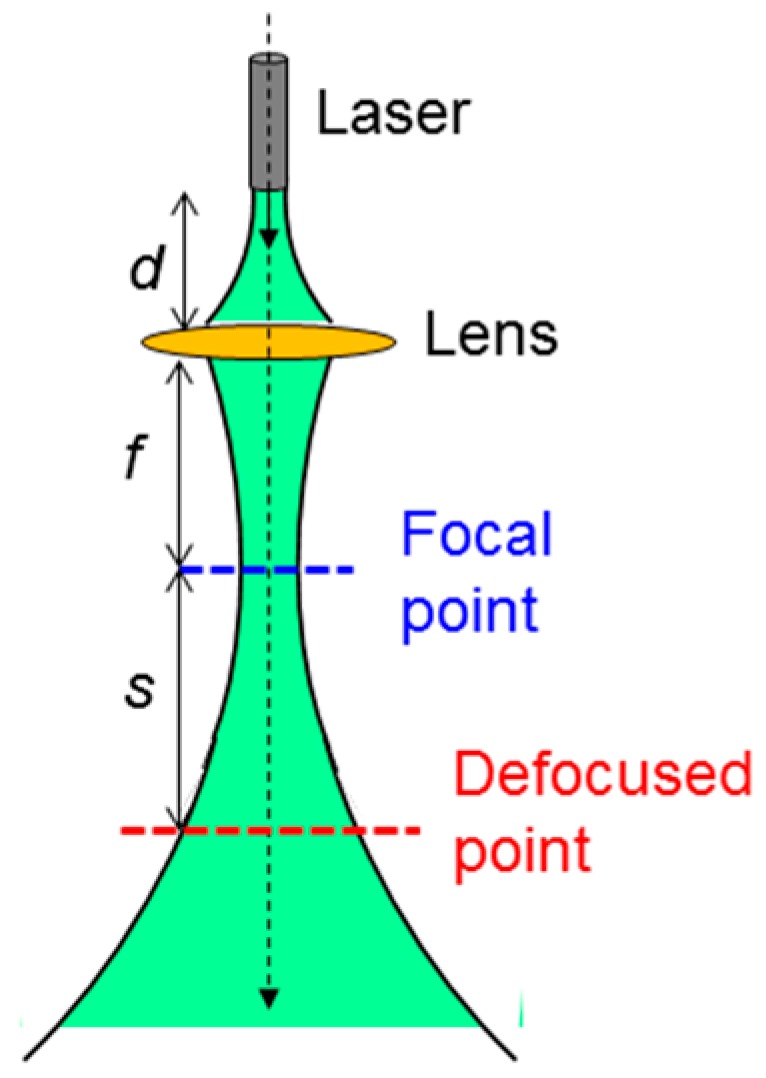
Schematic illustration of the setup for laser illumination onto cell samples. Cell wells were placed at the focal and defocused points for cell damage and perforation/preheating processes, respectively.

The experimental procedures and conditions are as follows: In the cases of NRI and NRI-AlPcS applications, NRI or NRI-AlPcS was first incubated with SAS cells for 12 h. Then, cell samples were washed or etched for the post-washout or post-etching condition, respectively. Next, the perforation or preheating process was applied by illuminating cell samples with the designated laser condition. After the perforation or preheating laser illumination, we waited 30 min for cell annealing and then applied the cell damage laser illumination with the designated conditions. Finally, trypan blue stain was applied for observing the cell damage area 4 h after the cell damage laser illumination. In the case of the NRI + AlPcS application, NRI was first incubated with SAS cells for 12 h. AlPcS of 10 µM in concentration was added to the cell samples after they were washed or etched for the post-washout or post-etching condition, respectively. The laser illumination for perforation or preheating was applied right after the addition of AlPcS to the cell samples. The rest of the procedures were the same as those in the cases of NRI or NRI-AlPcS application. The laser illumination setup is shown in Figure 2. Here, one can see that a convex lens was used to focus the laser beam. For damaging cells incubated in a well, the well was placed at the focal point. For producing the perforation or preheating effects, the cell well is placed at the defocused point. The distance between the laser head and the lens is designated as *d*. The focal length of the lens is *f*. The distance between the focal point and the designated defocused point is *s*. In using the 660-nm cw laser, *d* and *f* were 18 and 6.7 cm, respectively. When we used the 1064-nm fs (cw) laser, *d*, *f*, and *s* were 4.1, 2.7, and 7.4 (3.4) cm, respectively. The laser illumination experiments were undertaken at room temperature in the atmosphere. No temperature control was applied. The laser illumination conditions, including power, beam size, (average) peak intensity, illumination duration, and (average) peak fluence of the used 1064-nm fs laser, 1064-nm cw laser, and 660-nm cw laser for cell perforation, preheating, and damage are listed in Table 1. Here, we can see that in using the 1064-nm fs laser for cell perforation, the average peak fluence was only ≈3% of the peak fluence of the 1064-nm cw laser for cell preheating. Also, the used average peak fluence of the 1064-nm fs laser for cell damage was ≈67 times that for cell perforation. However, the used peak fluence of the 1064-nm cw laser for cell damage was only ≈2.87 times that for cell preheating. The used peak fluence of the 660-nm cw laser for cell damage was significantly lower than those of the 1064-nm fs and cw lasers, indicating that the required laser fluence for generating the PD effect was significantly lower than those for producing the PT effect.

## 3. Perforation and Preheating

**Figure 3:**
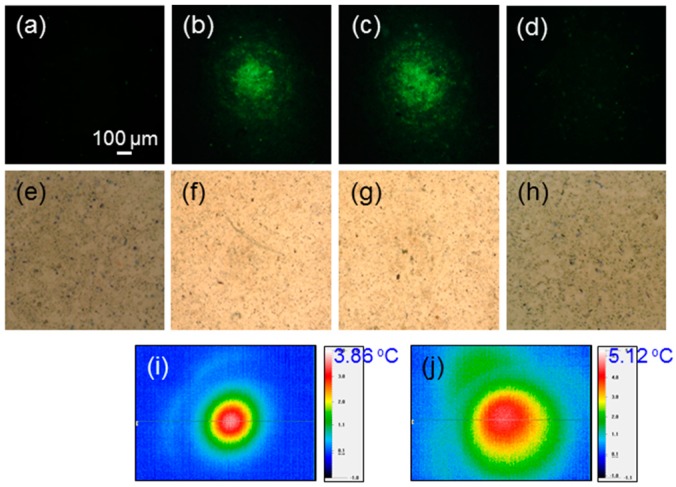
(**a**–**d**): Fluorescent microscopy images of cells incubated with Au NRIs and green-emitting Lucifer yellow under the conditions of no laser illumination, illuminations of the defocused 1064-nm fs laser for 30 and 60 s, and illumination of the defocused, lower-power 1064-nm cw laser for 10 min, respectively, under the post-washout condition. (**e**–**h**): Trypan-blue-stained cell images around the laser illumination spots 4 h after laser illuminations corresponding to parts (**a**–**d**), respectively. (**i**,**j**): Temperature-change mapping images in the perforation (30-s illumination) and preheating processes, respectively, under the post-washout condition. The shown numbers in °C indicate the maximum temperature changes under individual conditions.

To implement cell perforation or preheating, we needed to find a suitable laser intensity for causing the perforation or preheating effect, but not leading to a permanent damage or death of cell. Figure 3a–d show the fluorescent microscopy images of cells incubated with Au NRI and green-emitting Lucifer yellow under the conditions of no laser illumination (Figure 3a), illuminations of the defocused 1064-nm fs laser for 30 (Figure 3b) and 60 (Figure 3c) sec (see the laser conditions in row 2 of Table 1 except the illumination duration), and illumination of the defocused, lower-power 1064-nm cw laser for 10 min (Figure 3d) (see the laser conditions in row 3 of Table 1), respectively, under the post-washout condition. These images were used for showing the fluorescence distribution of Lucifer yellow for us to understand whether Lucifer yellow had been internalized by cells through the perforation and preheating processes. Without laser illumination, Lucifer yellow could not be internalized, as shown in Figure 3a. As shown in Figure 3b,c, with the illumination of the defocused 1064-nm fs laser, cells were perforated for Lucifer yellow to be internalized such that green fluorescence could be observed. With a longer fs-laser illumination, the green fluorescence became stronger and its distribution was broader. However, as shown in Figure 3d, with the illumination of the defocused 1064-nm cw laser, the heating effect could not help with Lucifer yellow uptake and no green fluorescence could be seen. The heating effect could increase the permeability of the cell membrane. However, such a permeability increase did not allow a large molecule, like Lucifer yellow, to enter a cell. The perforation or preheating process was designed only for increasing the cell uptake of Au NPs, photosensitizer, and other drugs, but not causing permanent damage or death of the cell. In Figure 3e–h, we show the trypan-blue-stained cell images around the laser illumination spots 4 h after laser illuminations, corresponding to the cases of Figure 3a–d, respectively. Here, one cannot see any stained spot, indicating that the perforation or preheating process does not cause cell death. Figure 3i,j show the temperature-change mapping images in the perforation (30-s illumination) and preheating processes, respectively, under the post-washout condition. The labelled numbers in °C indicate the maximum temperature changes under individual conditions. One can see that the perforation process leads to only <4 °C temperature change. The temperature distribution on a cell sample was measured with a thermal imaging system (P640RD-20–16 µm, Ching Hsing Computer-Tech Ltd., Taipei, Taiwan). The spatial resolution of this imaging system is 16 µm.

**Figure 4:**
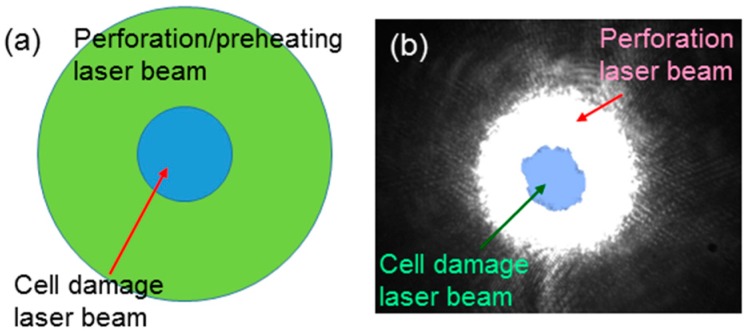
(**a**): Schematic demonstration of the overlap of the cell damage laser beam of a relatively smaller beam size with the perforation or preheating laser beam of a larger beam size. (**b**): CCD images of the defocused beam of the 1064-nm fs laser used for perforation (white region) and the focused beam of the same laser for cell damage (light blue region).

To observe the influence of cell perforation or preheating on cell damage, we needed to make sure that the illumination laser spot for cell damage overlapped that for perforation or preheating. We carefully aligned the two laser beams such that their beam centers coincided with each other. In other words, the cell damage laser beam of a relatively smaller beam size was covered by the perforation or preheating laser beam of a larger beam size, as schematically shown in Figure 4a. Figure 4b shows the charge-coupled device (CCD) images of the defocused beam of the 1064-nm fs laser used for perforation (white region) and the focused beam of the same laser for cell damage (light blue region). Here, we can see that the area of the perforation laser beam covered that of the cell damage laser beam well. The laser illumination conditions for the perforation and preheating processes for the following cell damage experiments were fixed at those shown in rows 2 and 3, respectively, of Table 1. The laser illumination conditions for cell damage were fixed at those shown in rows 4–6 of Table 1.

## 4. Cancer Cell Damage Areas

**Figure 5:**
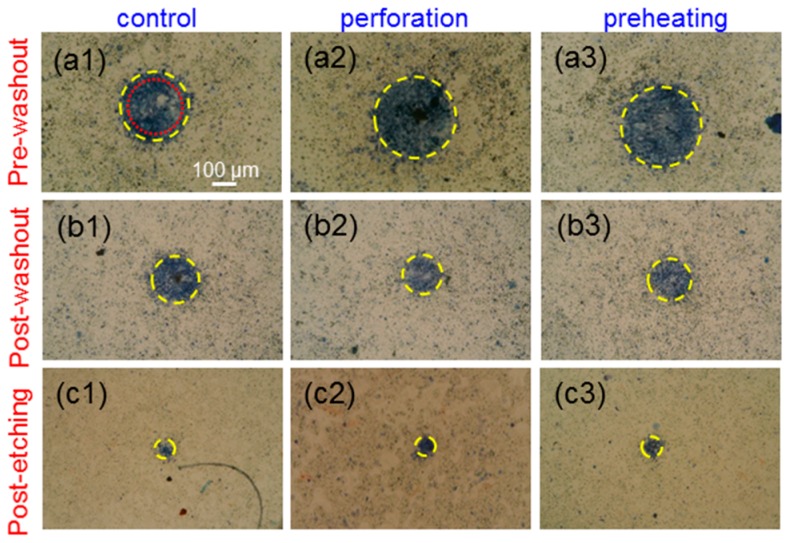
(**a1**–**a3**): Images of trypan blue stained cells incubated with NRI + AlPcS under the pre-washout condition in the cases of no pre-treatment (control), perforation, and preheating, respectively, when cell samples were illuminated by the focused 1064-nm cw laser. (**b1**–**b3**) and (**c1**–**c3**) show the images similar to parts (**a1**–**a3**), respectively, under the post-washout and post-etching condition, respectively.

**Figure 6:**
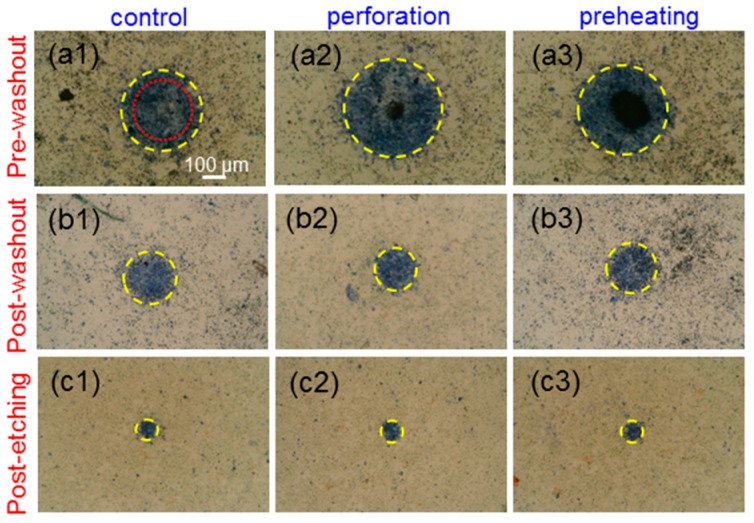
(**a1**–**c3**): Results similar to Figure 5(**a1**–**c3**), respectively, when cell samples were illuminated using the focused 1064-nm fs laser.

**Figure 7:**
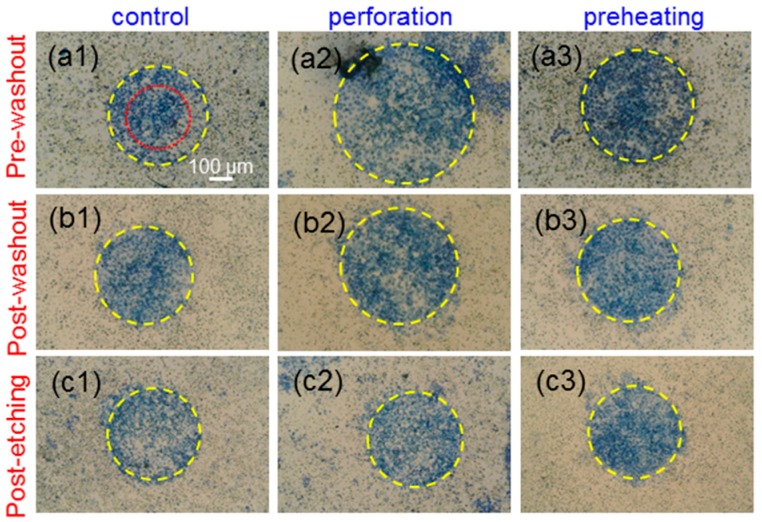
(**a1**–**c3**): Results similar to Figure 5(**a1**–**c3**), respectively, when cell samples were illuminated using the 660-nm laser.

Figure 5(a1–a3) show the images of trypan-blue-stained cells incubated with Au NRI + AlPcS under the pre-washout condition in the cases of no pre-treatment (control), perforation, and preheating, respectively, when cell samples were illuminated by the focused 1064-nm cw laser to cause cell damage through the PT effect. The yellow dashed circles were drawn to best fit the circular trypan blue stained areas. The red dotted circle in Figure 5(a1) indicates the radius at which laser intensity drops to the 36.8% (10^−1^) level from the central peak. Figure 5(b1–b3,c1–c3) show the images similar to Figure 5(a1–a3), respectively, under the post-washout (post-etching) condition. Under the pre-washout condition, either perforation or preheating led to a larger cell damage area. The perforation process was more effective in causing cell damage when compared with preheating. However, under the post-washout condition, with perforation or preheating, the cell damage area became slightly smaller. Under the post-etching condition, the perforation or preheating process made a negligibly small change in cell damage area. Figure 6(a1–c3) show the results similar to Figure 5(a1–c3), respectively, when cell samples were illuminated using the focused 1064-nm fs laser to cause cell damage through the PT effect. Although AlPcS was also taken up by cells in this NRI + AlPcS incubation case, because AlPcS was not linked with Au NRIs, the illumination of the 1064-nm fs laser could not effectively excite the two-photon absorption of AlPcS for generating the PD effect. The difference in AlPcS linkage caused different cell damage results between the NRI-AlPcS and NRI + AlPcS cases. As shown in Figure 5(a1–c3) and Figure 6(a1–c3), in the case of NRI + AlPcS incubation, the variation trends of cell damage area under the illuminations of the 1064-nm cw and fs lasers were the same. However, the cell damage area under the illumination of the fs laser was larger than the corresponding area under the illumination of the cw laser. This result indicated that the fs laser illumination was more effective in generating the PT effect, when compared with the cw laser illumination, even though the used average peak fluence of the fs laser was lower than the peak fluence of the cw laser. Figure 7(a1–c3) show the results similar to Figure 5(a1–c3), respectively, when cell samples were illuminated by the 660-nm laser for causing cell damage through the PD effect. Here, under the pre-washout condition, both the perforation and preheating processes led to significant increases of cell death area, where the perforation process was more effective in enhancing cell damage efficiency. Different from the cases of 1064-nm cw and fs laser illuminations, under the post-washout condition, both the perforation and preheating processes also resulted in increases of the cell damage area. Under the post-etching condition, the perforation process caused a slight increase in cell damage area, although the preheating process did not significantly change the cell damage area.

Cell damage images stained using trypan blue similar to Figure 5, Figure 6 and Figure 7 were also obtained in the cases of NRI and NRI-AlPcS incubations when the cell samples were illuminated using the 1064-nm cw and fs lasers, and the 660-nm laser under the pre-washout, post-washout, and post-etching conditions. Based on Equation (2), from the radius (*r_th_*) of a circular cell damage area, given *P* and *r*_0_ (see Table 1), we could evaluate the laser intensity (*I_th_*). This laser intensity is referred to as the threshold intensity for causing a cell damage condition as stained by trypan blue. By multiplying the threshold intensity with laser illumination duration, we can obtain the threshold fluence.

## 5. Cell Damage Threshold Fluences

**Figure 8:**
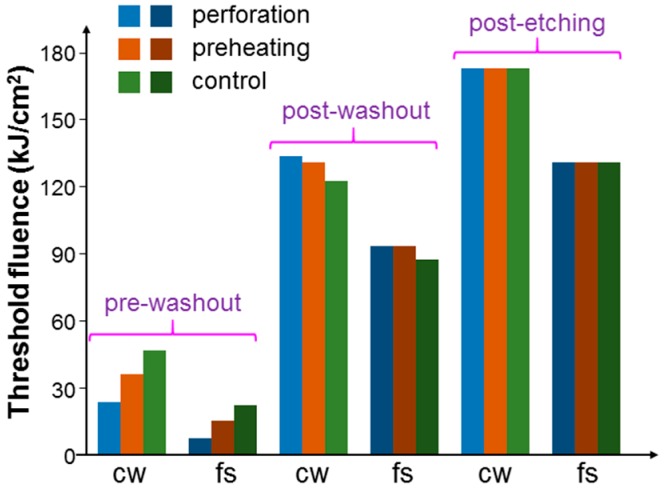
Threshold fluences with the illuminations of the focused 1064-nm cw and fs lasers under the pre-washout, post-washout, and post-etching conditions when cells were incubated with NRI only.

Figure 8 shows the threshold fluences with the illuminations of the 1064-nm cw and fs lasers under the pre-washout, post-washout, and post-etching conditions when cells were incubated with NRI only. Here, one can see that with the fs laser illumination, the threshold fluences were always lower, when compared to the results with the cw laser illumination, indicating that with the same average power of an fs laser, the fs laser could produce a more effective PT effect. Under the pre-washout condition, the perforation process was more effective in increasing the cell uptake of Au NRIs, when compared with the preheating process. However, under the post-washout condition, either perforation or preheating led to a slightly higher threshold fluence for cell damage. This result was due to the internalization of the NRIs, which were originally adsorbed onto the cell membrane during the perforation or preheating process. According to our previous study results [23,24,25], NRIs adsorbed onto the cell membrane could more effectively cause cell necrosis through the PT effect when compared with internalized NRIs. Under the post-washout condition, in either the perforation or preheating process, the adsorbed NRIs were internalized such that the PT effect became weaker. Under the post-etching condition, the threshold fluences were about the same among the cases of control, perforation, and preheating. It is noted that under the pre-washout condition, both the suspended (NRIs not taken up by cells) and adsorbed Au NRIs could contribute to the perforation or preheating effect. Under the post-washout condition, the perforation effect relied on the Au NRIs adsorbed onto the cell membrane. Under the same condition, the preheating effect may have had the contributions from both adsorbed and internalized Au NRIs. Then, under the post-etching condition, we expected a negligibly weak perforation effect because there was no Au NRI outside the cells. In this situation, the internalized Au NRIs could still produce the preheating effect for increasing the permeability of the cell membrane. However, because there was no Au NRI outside cells, the increase of the membrane permeability could only possibly result in the exocytosis of the originally internalized Au NRIs. Nevertheless, the almost equal threshold fluences in the cases of control, perforation, and preheating indicated that the exocytosis effect was weak.

**Figure 9:**
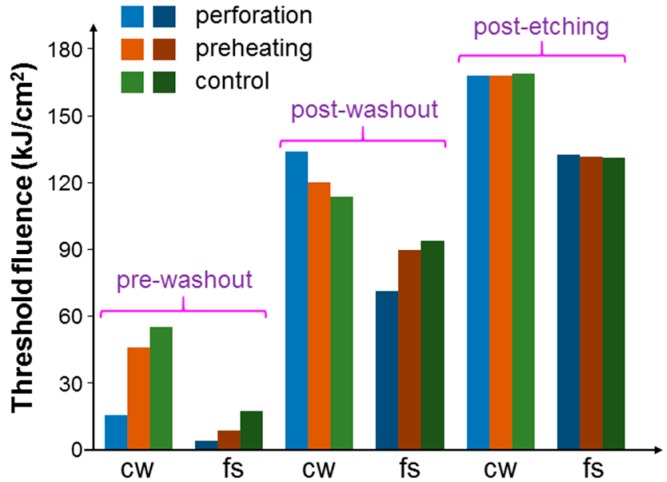
Threshold fluences similar to those in Figure 8 when cells were incubated with NRI-AlPcS.

**Figure 10:**
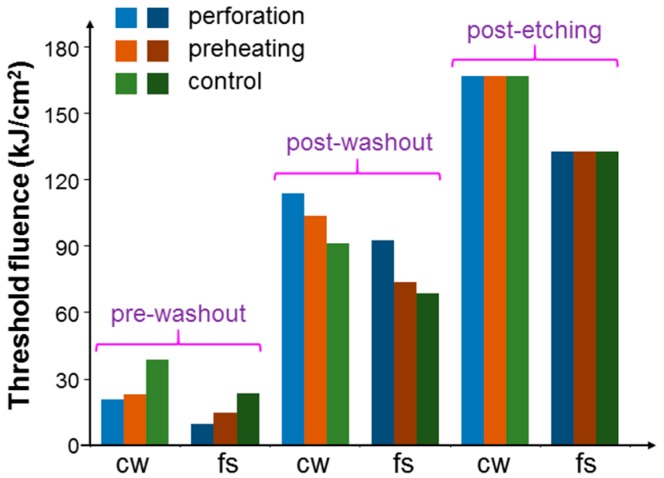
Threshold fluences similar to those in Figure 8 when cells were incubated with NRI + AlPcS.

Figure 9 shows the threshold fluences similar to those in Figure 8 when cells were incubated with NRI-AlPcS. Here, the variation trends of threshold fluence were the same as those in Figure 8 except the situation of fs laser illumination under the post-washout condition. In this situation, different from the result of incubating NRI only, the incubation of NRI-AlPcS could lead to the PD effect, besides the PT effect, under the fs laser illumination. Hence, although the NRI-AlPcS complexes adsorbed onto cell membrane were internalized, and hence the PT effect could be reduced, the increased PD effect of the newly internalized NRI-AlPcS complexes inside cells could enhance the overall cell damage effect. The generated ROS inside the cells was more effective in damaging cells, when compared with that on cell membrane. Therefore, the threshold fluence was reduced with either the perforation or preheating process. Figure 10 shows the threshold fluences similar to those in Figure 8 when cells were incubated with NRI + AlPcS. The variation trends in Figure 10 were the same as those in Figure 8. In other words, in the situation of fs laser illumination under the post-washout condition, either perforation or preheating led to an increase of the threshold fluence. In this situation, different from the result of incubating NRI-AlPcS, the incubation of NRI + AlPcS could not lead to a PD effect under the fs laser illumination because of the large distance between NRI and AlPcS. Therefore, similar to the situation of incubating NRI only, the reduced PT effect resulted in increased threshold fluence with either perforation or preheating.

**Figure 11:**
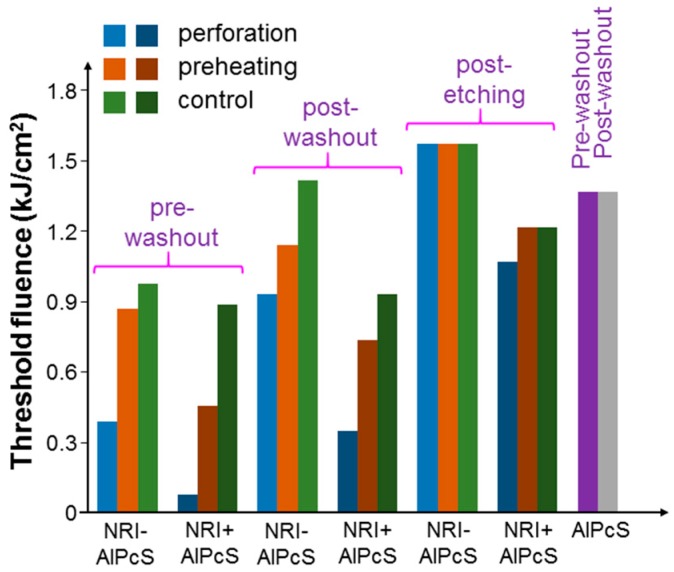
Results of threshold fluence under the pre-washout, post-washout, and post-etching conditions with the illumination of the 660-nm laser when cells were incubated with NRI-AlPcS and NRI + AlPcS. Here, we also show the threshold fluences under the pre-washout and post-washout conditions with the illumination of the 660-nm laser when AlPcS alone (10 µM) was incubated with cells.

Figure 11 shows the results of threshold fluence under the pre-washout, post-washout, and post-etching conditions with the illumination of the 660-nm laser when cells were incubated with NRI-AlPcS and NRI + AlPcS. Here, under the pre-washout and post-washout conditions, either perforation or preheating could increase the AlPcS uptake (either linked with NRI or not) and hence cell damage efficiency. In particular, more AlPcS molecules were taken up when NRI and AlPcS were incubated with cells separately (the NRI + AlPcS case). This result could be attributed to the high AlPcS concentration (10 µM) used for incubating with cells. The AlPcS concentration in the NRI-AlPcS solution could be lower than 10 µM. Also, the cell uptake pathways for NRI-AlPcS and AlPcS alone could be different. Under the post-washout condition, the perforation or preheating process could internalize the NRI-AlPcS complexes and AlPcS molecules adsorbed onto the cell membrane for enhancing the PD effect under the illumination of the 660-nm laser. As shown in Figure 8, Figure 9, Figure 10 and Figure 11, perforation could always reduce the threshold fluences more effectively than preheating. In Figure 11, the differences of threshold fluence between the perforation and preheating effects become even larger. In Figure 8, Figure 9 and Figure 10, under the condition of post-etching, neither perforation nor preheating significantly changed the threshold fluence. However, under the 660-nm laser illumination, in the case of NRI + AlPcS incubation, perforation could increase AlPcS uptake and hence reduce the threshold fluence. This was because KI/I_2_ only etched NRIs, while AlPcS molecules could still attach onto the cell membrane after KI/I_2_ etching. Those AlPcS molecules could be internalized during the perforation process for reducing the threshold fluence even though the perforation effect could be negligibly weak under the post-etching condition. It is interesting to note that this process was ineffective through preheating. In Figure 11, we also show the threshold fluences under the pre-washout and post-washout conditions with the illumination of the 660-nm laser when AlPcS alone (10 µM) was incubated with cells. Here, about the same threshold fluences under the pre-washout and post-washout conditions confirmed that AlPcS outside cells did not cause a significant PD effect. It is noted that the threshold fluence of incubating AlPcS only was larger than those of the control cases with NRI + AlPcS (the same AlPcS concentration at 10 µM) incubation under the pre-washout, post-washout, and post-etching conditions. These comparison results indicated that with separate NRI co-incubation with cells, AlPcS uptake was more effective, even though perforation or preheating was not applied. The mechanism behind such a phenomenon deserves further investigation.

## 6. Discussions: Increase of Cell Uptake Efficiency

**Figure 12:**
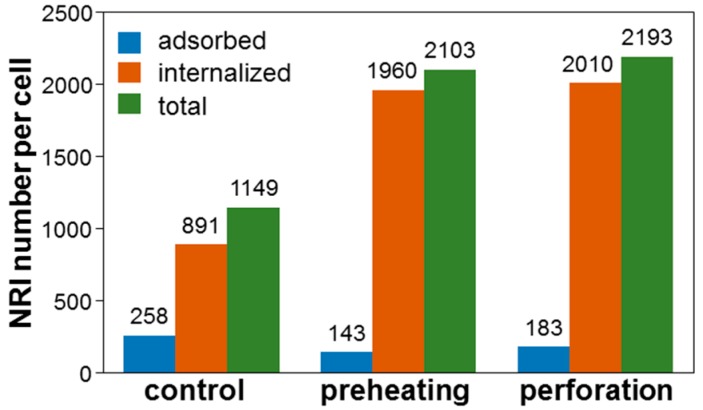
ICP-MS data for the number of NRIs per cell through the perforation and preheating processes when NRI-AlPcS was incubated with cells.

To confirm the increase of cell uptake efficiency, we used the techniques of ICP-MS and cell flow cytometry for evaluating the Au NRI and AlPcS concentrations in cells with and without perforation or preheating. For such evaluations, the cell illumination condition in a well needed to be as uniform as possible such that the cells in the whole well could be used for measurement. Because the used laser beam size for either perforation or preheating was not large enough for covering the whole cell well, we needed to scan the laser beam over the well with the same laser power and beam size as those shown in row 2 or 3 of Table 1. The scan pattern was controlled by an electrically driven two-dimensional translational stage. The line-scan speed was 0.1 mm/s and the translation distance for the next line-scan was 0.2 mm. Figure 12 shows the ICP-MS data of the number of NRIs per cell when NRI-AlPcS was incubated with cells. Here, one can see that after perforation or preheating, the number of internalized NRIs per cell was significantly increased. However, the adsorbed number of NRIs per cell was reduced. The number of NRIs taken up per cell through the perforation (preheating) process was increased by 90.9 (83.0)%. The cell uptake amounts of NRI-AlPcS were similar in both preheating and perforation cases, although that in the case of perforation was slightly larger (90.9% vs. 83.0%). These results may suggest that perforation can more effectively increase NP uptake when compared with preheating. This trend is consistent with the cell damage results reported earlier.

**Figure 13:**
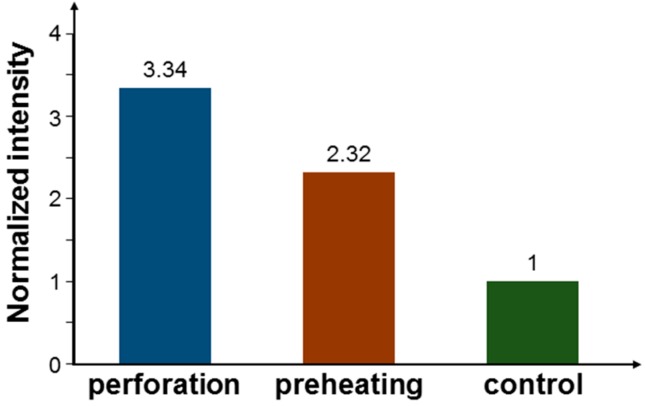
Normalized AlPcS fluorescence intensities based on flow cytometry measurement through the perforation and preheating processes when NRI-AlPcS is incubated with cells.

Because samples could be easily aluminium-contaminated, it was unreliable to use ICP-MS for evaluating the AlPcS molecule number in a sample through the measurement of aluminium content. Therefore, we used the flow cytometry technique to observe the fluorescence intensity of AlPcS in cells for evaluating the effects of perforation and preheating on cell uptake of AlPcS when NRI-AlPcS was incubated with cells. Under the same laser scanning condition as mentioned earlier, we obtained the normalized AlPcS fluorescence intensity results shown in Figure 13. Here, one can see that through the perforation and preheating processes, the uptake amounts of AlPcS molecules increased by 234 and 132%, respectively. Because the cell illumination conditions based on laser scanning were different from those with fixed laser beams, which were used for obtaining the cell damage results shown in Figure 8, Figure 9, Figure 10 and Figure 11, the ICP-MS and flow cytometry data could not provide us with the same numbers of NRIs and AlPcS per cell as those for cell damage. However, the variation trends in Figure 12 and Figure 13 do indicate the effects of increasing cell uptake efficiency through the perforation and preheating processes.

Because we needed many NRI and cell samples, and quite a long time to obtain a data set, performing multiple experiments under the same conditions for obtaining statistics or error bars was difficult. Therefore, in Figure 8, Figure 9, Figure 10, Figure 11, Figure 12 and Figure 13 we cannot show error bars or other statistics data. Without error bars, we may not have strong confidence in the demonstrated variation trends. However, they do illustrate the general trends and provide us with useful information. In particular, our observations based on different measurements, including cell damage calibration, ICP-MS, and flow cytometry, were all consistent, indicating that the observed variation trends were reliable. In other words, the results from different measurements support each other, just like multiple experiments under the same condition for forming statistics support each other. A repeated experiment may not produce exactly the same results in terms of quantity; however, the variation trends should be reproduced. 

It is noted that under strong heating, an Au nanorod (NR) can turn into a sphere-like NP [28,29]. This is so because the NR is small (typically several tens nm in length and several to ten something nm in width), compared with our NRI of >100 nm in dimension, and hence can be easily deformed through heating. Also, because of the weak scattering nature of an NR, the major function of its LSP resonance is absorption and hence heating, leading to a strong PT effect. On the contrary, the larger size and its circular symmetry of NRI make its deformation more difficult during heating. In particular, because of the strong scattering behavior of NRI, the heating effect of NRI is not as strong as that of NR. Therefore, under the used laser illumination conditions, the morphology of NRIs is not significantly changed. In certain experiments, we recycled NRIs and found that their LSP resonance behavior was not significantly changed, indicating that the morphology of NRIs was not significantly changed after laser illumination. It was also noted that the threshold levels of power density or fluence for causing cell damage through the PT effect reported above were in the same order of magnitude as those in literature [30,31]. However, such a level is still too high for clinical application. More research efforts are needed to reduce the required power density level for effectively producing PT effects.

## 7. Conclusions

In summary, we have used the methods of cell perforation and preheating for increasing cell uptake efficiencies of Au NRIs, which had the LSP resonance wavelength around 1064 nm, and photosensitizer AlPcS to enhance the cell damage efficiency through the PT and PD effects. The perforation and preheating effects were generated by illuminating a defocused 1064-nm fs laser and a defocused 1064-nm cw laser, respectively. Cell damage was produced by illuminating cell samples with a focused 1064-nm cw laser for producing the PT effect, a focused 1064-nm fs laser for producing both PT and PD effects, and a focused 660-nm cw laser for producing the PD effect. The results showed that under various conditions of pre-washout and post-washout, with either perforation or preheating process, cell uptake and hence cell damage efficiencies could be enhanced. Under the current experimental conditions, perforation could be more effective in the aforementioned functions when compared with preheating.

## Figures and Tables

**Table 1:** Laser conditions for generating the cell perforation, preheating, and damage effects. For the fs laser, the time-average values are given.

	Power, *P* (mW)	Beam Size, *r*_0_ (µm)	(Average) Peak Intensity (W/cm^2^)	Illumination Duration (min)	(Average) Peak Fluence (kJ/cm^2^)
Defocused 1064-nm fs laser for perforation	130	234	75.6	0.5	2.27
Defocused 1064-nm cw laser for preheating	80	152	110.9	10	66.54
Focused 1064-nm fs laser for cell damage	130	128	254.6	10	152.76
Focused 1064-nm cw laser for cell damage	130	114	318.4	10	191.04
Focused 660-nm cw laser for cell damage	10	135	17.5	10	10.50
